# Defining the Roles of IFN-γ and IL-17A in Inflammation and Protection against *Helicobacter pylori* Infection

**DOI:** 10.1371/journal.pone.0131444

**Published:** 2015-07-13

**Authors:** Louise Sjökvist Ottsjö, Carl-Fredrik Flach, Staffan Nilsson, Rene de Waal Malefyt, Anna K. Walduck, Sukanya Raghavan

**Affiliations:** 1 Department of Microbiology & Immunology, University of Gothenburg, Gothenburg, Sweden; 2 Department of Mathematical Sciences, Chalmers University of Technology, Gothenburg, Sweden; 3 Department of Immunology, Merck Research Laboratories, Palo Alto, California, United States of America; 4 School of Applied Sciences, RMIT University, Bundoora, Victoria, Australia; Institut Pasteur Paris, FRANCE

## Abstract

CD4^+^ T cells have been shown to be essential for vaccine-induced protection against *Helicobacter pylori* infection. However, the effector mechanisms leading to reductions in the gastric bacterial loads of vaccinated mice remain unclear. We have investigated the function of IFN-γ and IL-17A for vaccine-induced protection and inflammation (gastritis) using IFN-γ-gene-knockout (IFN-γ^-/-^) mice, after sublingual or intragastric immunization with *H*. *pylori* lysate antigens and cholera toxin. Bacteria were enumerated in the stomachs of mice and related to the gastritis score and cellular immune responses. We report that sublingually and intragastrically immunized IFN-γ^-/-^ mice had significantly reduced bacterial loads similar to immunized wild-type mice compared to respective unimmunized infection controls. The reduction in bacterial loads in sublingually and intragastrically immunized IFN-γ^-/-^ mice was associated with significantly higher levels of IL-17A in stomach extracts and lower gastritis scores compared with immunized wild-type mice. To study the role of IL-17A for vaccine-induced protection in sublingually immunized IFN-γ^-/-^ mice, IL-17A was neutralized *in vivo* at the time of infection. Remarkably, the neutralization of IL-17A in sublingually immunized IFN-γ^-/-^ mice completely abolished protection against *H*. *pylori* infection and the mild gastritis. In summary, our results suggest that IFN-γ responses in the stomach of sublingually immunized mice promote vaccine-induced gastritis, after infection with *H*. *pylori* but that IL-17A primarily functions to reduce the bacterial load.

## Introduction

*Helicobacter pylori* infection in the stomach is characterised by gastritis with both active (neutrophils) and chronic (T and B cells) components [1]. Although a robust *H*. *pylori*-specific immune response can be detected in the circulation and stomach of infected individuals, it is insufficient to eradicate the infection [2]. Furthermore, epidemiological studies have shown that the immune response to a previous infection with *H*. *pylori* does not protect against reinfection by subjects in whom the infection has been eradicated by antibiotic therapy [3–5]. Vaccination with *H*. *pylori* antigens and an adjuvant can potentially enhance the infection-induced immune responses, resulting in a reduction in gastric bacterial loads [6, 7] and also protect against reinfections [3] in animal models.

Indeed, studies in a mouse model of *H*. *pylori* infection have demonstrated that prophylactic sublingual (under the tongue; SL) [8] or intragastric (IG) [5] immunization with *H*. *pylori* antigens and the mucosal adjuvant cholera toxin (CT) reduced the gastric bacterial loads and that this phenomenon was dependent on the presence of CD4^+^ T cells rather than antibodies [9–11]. Furthermore, the immunization polarized the CD4^+^ T helper cells to produce enhanced interferon-γ (IFN-γ) and interleukin-17A (IL-17A) in the stomach when compared with unimmunized mice [8, 12]. However, in spite of a strong influx of CD4^+^ T cells, B cells, eosinophils, mast cells and dendritic cells in the stomach of vaccinated mice post-challenge which manifests as severe gastritis, the infection is rarely eradicated [12–14]. Clearly, we need to improve our understanding of the differences between infection and vaccination induced gastritis and the mechanisms by which this extra cellular bacterium can be eliminated.

Previous reports on the role of IFN-γ in the vaccine-induced protection against *H*. *pylori* infection have been contradictory. Using IFN-γ^-/-^ mice, one study reported the complete dependence on IFN-γ for protection after IG immunization at two weeks post challenge [15], whereas another study showed no dependence of IFN-γ for protection after intranasal (IN) immunization at four weeks post challenge [16]. The same antigens (*H*. *pylori* lysate antigens) and mucosal adjuvant (cholera toxin; CT) were used in both studies. Furthermore, the studies demonstrated that post-immunization gastritis was dampened in intragastrically but not intranasally immunized IFN-γ^-/-^ mice, although the differing times after *H*. *pylori* challenge may also play a role [15, 16]. We have previously shown that the *in vivo* neutralization of IFN-γ at the time of challenge in wild-type sublingually immunized mice did not abrogate protection against *H*. *pylori* infection. The effect of this treatment on post-immunization gastritis, however, was not studied [17]. Thus, the importance of IFN-γ in sublingually vaccinated mice for protection against *H*. *pylori* infection and/or post-immunization gastritis is currently unknown.

The contradictory reports on the role of IFN-γ in the protection against *H*. *pylori* infection using knockout mice might be explained by the presence of IL-17A in the stomach [8, 17–19]. As described previously, IL-17A has been detected in the stomach of wild-type vaccinated mice [17–19] and in *H*. *pylori*-infected individuals [20]. Inflammatory cytokines IL-1β, TGF-β, IL-6 and IL-23, which promote IL-17A secretion from CD4^+^ T cells (Th17 differentiation), have all been shown to be enhanced in the stomachs of *H pylori*-infected individuals [20]. Additionally, the vaccine-induced protection against *H*. *pylori* infection in mice was associated with an increase in IL-17A; presumably this mediates the recruitment of neutrophils by inducing chemokine secretion in various cell populations. IL-17A and IL-22 can also induce the secretion of antimicrobial agents like β-defensins, lactotransferrin and lipocalin-2 from epithelial cells and neutrophils [21, 22]. We have previously reported that *in vivo* neutralization of IL-17A abolished protection in immunized WT mice [17]. Further, Delyria et al. showed that neutrophils were required for protection [17, 19]. These reports support a critical role for IL-17A in protective mechanisms.

The aim of the current study was to define the function of IFN-γ and IL-17A in the vaccine-induced protection against *H*. *pylori* infection and post-immunization gastritis after SL immunization. We report that both IFN-γ^-/-^ and wild-type mice immunized by the SL or IG route show a significant reduction in the gastric bacterial loads compared to unimmunized controls. Immunized IFN-γ^-/-^ mice had a significantly elevated IL-17A secretion in the stomach, and lower gastritis scores compared with immunized wild-type mice. The role of IL-17A in protection was further illustrated by the observation that *in vivo* neutralization of IL-17A in sublingually immunized IFN-γ^-/-^ mice abrogated the protection against *H*. *pylori* infection. We conclude that IL-17A rather than IFN-γ can be important for the reductions in *H*. *pylori* colonization in the stomach of sublingually immunized mice.

## Materials and Methods

### Animals

Six-to-eight-week-old, specific-pathogen-free, female C57BL/6 wild-type mice were purchased from Taconic (Denmark). Age-matched IFN-γ-deficient (IFN-γ^-/-^) mice on a C57BL/6 background [23] were bred at the Laboratory for Experimental Biomedicine (EBM), University of Gothenburg, and all mice were housed in microisolators for the duration of the study. This study was carried out in strict accordance with the recommendations in the Guide for the Care and Use of Laboratory Animals of the Swedish Board of Agriculture. The study was approved by the government animal ethics committee (Gothenburg; permit Number: 273/2011). All immunizations and infections were performed under anaesthesia (Isoflurane; Abbott Scandinavia AB, Solna, Sweden), and all efforts were made to minimize suffering.

### Cultivation of *H*. *pylori* SS1 used for infection

The *H*. *pylori* bacterial strain SS1 was cultured on Columbia iso agar plates for 3 days at 37°C under microaerophilic conditions and then transferred to liquid broth before the infection of mice, as previously described [8]. The optical density of the bacteria was adjusted to 1.5, corresponding to an infectious dose of approximately 2.5 x 10^9^ viable bacteria/ml [5].

### Preparation of *H*. *pylori* antigens

The *H*. *pylori* lysate antigens from the strain Hel 305 (CagA^+^, VacA^+^) was prepared as previously described in detail in [8, 24]. The prepared antigen was aliquoted and stored at -70°C. Aliquots of *H*. *pylori* lysate antigens for use in SL immunizations were freeze-dried and reconstituted to a protein concentration of 20 mg/ml to reduce the volume used for the immunizations. The same dose of antigen was used for both IG and SL immunizations. The *H*. *pylori* membrane protein (MP) antigen from strain Hel 305 (CagA^+^, VacA^+^) was prepared as previously described [25]. The antigen preparations were aliquoted and stored at -70°C until needed to coat the ELISA plates for the detection of serum antibody titres.

### Immunization and infection with *H*. *pylori* and neutralizing antibody treatment

Lyophilized CT from *Vibrio cholerae* (Sigma Aldrich, St. Louis, MO) was reconstituted in sterile distilled water to a concentration of 1 mg/ml. Groups of mice were immunized sublingually or intragastrically two times at a bi-weekly interval with 200 μg of *H*. *pylori* lysate antigens together with 10 μg of CT on each occasion. The immunizations were administered under deep anaesthesia (Isoflurane) by carefully placing 10 μl of *H*. *pylori* lysate antigens reconstituted in CT without bicarbonate buffer through a micropipette under the tongue (SL route) or by placing 300 μl of *H*. *pylori* lysate antigens together with CT in 3% sodium bicarbonate buffer directly into the stomach using a blunt feeding needle (IG route). Two weeks after the last immunization, the mice were challenged intragastrically with ~1 x 10^9^ live *H*. *pylori* SS1 bacteria. In some experiments, the neutralizing IL-17A antibody clone JL7.1D10 (rat IgG) (Merck Research Labs, Palo Alto, CA) [26] was injected intraperitoneally on days 5, 8 and 11 post-challenge into sublingually immunized and unimmunized IFN-γ^-/-^ mice [17]. Purified rat IgG antibody (Sigma) was used as an isotype control.

### Quantitative culture of *H*. *pylori* from the stomach

Two-three weeks post-infection, the mice were euthanized by an overdose of anaesthesia (Isoflurane) followed by cervical dislocation. The stomach was collected and the number of bacteria/stomach was determined by quantitative culture [27]. Briefly, one half of each stomach was homogenized in brucella broth using a tissue homogenizer (Ultra Turrax, IKA Laboratory Technologies, Staufen, Germany) and dilutions of the homogenates were plated on blood skirrow agar plates (BD (Becton Dickinson Biosciences), San Diego, CA). After 7 days of incubation at 37°C under microaerophilic conditions, *H*. *pylori* colonies were counted. The fold-change in bacterial counts was calculated as the ratio of the mean Log_10_ number of bacteria in the infected control mice to the Log_10_ number of bacteria in individual vaccinated mice.

### Serum antibody responses

Blood was collected from the axillary plexus immediately before the mice were sacrificed. Serum antibody titres were determined by enzyme-linked immunosorbent assay (ELISA) as previously described [8]. Briefly, ELISA plates were coated with the membrane antigen preparation of *H*. *pylori* strain Hel 305 (MP Hel 305) and left overnight at room temperature. Bound IgG antibodies in sera were detected using horseradish peroxidase (HRP)-coupled goat anti-mouse IgG (Jackson Immuno Research, West Grove, PA) secondary antibodies and the reaction with substrate *o*-phenylenediamine dihydrochloride (OPD), added together with H_2_O_2_. The enzymatic reaction was then stopped by adding 1 M sulfuric acid, and the absorbance at 490 nm was then measured using a spectrophotometer. The antibody titers are defined as the reciprocal serum dilution giving an absorbance of 0.4 above the background.

### Cellular immune responses

Single-cell lymphocyte suspensions were prepared from the spleens and mesenteric lymph nodes (MLN) of the different groups of mice. Briefly, cells were seeded (2 x 10^5^ cells per well) in the presence or absence of boiled *H*. *pylori* strain Hel 305 lysate antigens (4 μg/ml) and cultured for 72 hours in Iscove’s medium (Biochrome, Berlin, Germany) supplemented with 10% heat-inactivated foetal calf serum (Sigma), 50 μM 2-mercaptoethanol (Sigma), 1 mM L-glutamine (Biochrome) and 50 μg/ml gentamicin (Sigma) (Iscove’s complete medium) at 37°C in a 5% CO_2_ atmosphere. Supernatants were collected and stored at -70°C for subsequent cytokine analysis. The proliferation of the cells was determined as previously described [25]. Cells were pulsed with 1 μCi of [3H] thymidine (Amersham Bioscience, Buckinghamshire, United Kingdom) for the last 6 to 8 h of culture. The cellular DNA was collected with a cell harvester (Skatron) on glass fiber filters (Wallac) and assayed for ^3^H incorporation using a liquid scintillation counter (Beckman, LKB, Bromma, Sweden).

### Measurement of IFN-γ and IL-17A in saponin extracts of stomach tissue and cell culture supernatants

Stomach tissue extracts were prepared using a 2% saponin-PBS solution as previously described [27]. Briefly, the stomach was excised and stored in a PBS solution containing 2 mM phenylmethylsulfonylfluoride, 0.1 mg/ml of soybean trypsin inhibitor (Sigma), 0.05 mM EDTA, 1% bovine serum albumin and 0.05% Tween. The tissue samples were thawed and then permeabilized with saponin (Sigma) at a final concentration of 2% in PBS at 4°C over night. Stomach tissue was then centrifuged at 13,000 × *g* for 10 min and the supernatants were analyzed for cytokines. Levels of IFN-γ and IL-17A in the extracted stomach tissue and cell culture supernatant were analysed using the mouse cytometric bead array (CBA) flex set kit (BD Biosciences) according to the manufacturer’s instructions.

### Histology

Longitudinal strips from the entire stomach were taken, fixed in 4% phosphate-buffered formalin and then embedded in paraffin. Sections 6-μm thick were cut and stained with haematoxylin and eosin. The slides were then examined by light microscopy (100x), and the extent of gastritis was graded as previously described [28]. The samples were “blinded” and given grades for tissue damage and cellular infiltration as follows: Grade 0–1, normal gastric mucosa that contained few lymphocytes scattered throughout the submucosa; Grade 2, small aggregates containing three to four layers of cells in the mucosa or sparse infiltrates of cells in the submucosa covering ~5% of the section; Grade 3, frequent and larger infiltrates extending into the mucosa; Grade 4, infiltrates spanning half to the entire width of the mucosa; Grade 2–5, partial or complete (grade 6) obliteration of parietal and chief cells with hyperplasia of mucous and epithelial cells.

### Cytokine staining and flow cytometric analysis

Spleen and MLN mononuclear cells were isolated and single-cell suspensions were prepared and live cells counted under a microscope. For the spleen an extra cell lysing step was carried out to remove the red blood cells (BD Biosciences). For cytokine staining, the cells were stimulated with 20 ng/ml phorbol 12-myristate 13-acetate (PMA, Sigma) and 1 μg/ml ionomycin (Sigma) in Iscove’s complete medium for 2 h at 37°C in a 5% CO_2_ atmosphere. After 2 h, 10 μg/ml Brefeldin A (Sigma) was added to the cells and left for an additional 2 h at 37°C in a 5% CO_2_ atmosphere. Surface or intracellular staining was performed using anti-mouse CD3-PerCP, CD4-Alexa Fluor 700, CD8-v450, NK1.1-PE (all BD Biosciences), IL-17A-FITC (eBioscience, San Diego, CA) and Live/Dead Fixable Aqua Dead Cell Stain Kit (Life Technologies, Carlsbad, CA). Stained cells were acquired using LSR-II Flow Cytometer and FACSDiva software (BD Biosciences), and subsequent analyses were conducted using FlowJo software (FlowJo, Treestar, Ashland, OR).

### RNA isolation

The stomach was excised and dissected along the greater curvature. Any loose stomach contents were removed by washing in PBS. One longitudinal strip including the corpus and antrum were cut and placed directly into RNAlater and stored at -70°C. For RNA isolation, the tissue was thawed and transferred to RLT lysis buffer and homogenized using a Tissue Lyser II. Total RNA was extracted using RNeasy mini kit (Qiagen) and then stored at -70°C. RNA purity and concentration was measured using the fluorospectrometer Nanodrop ND-1000.

### cDNA preparation and real-time PCR array

RNA (2 μg) was reverse transcribed into cDNA using the Quantitect kit (Qiagen). Pooled cDNA (2 μg) from individual mice in a group were run in a 96-well array plates (Mouse antibacterial response array, SA Biosciences/Qiagen) according to manufacturer´s instruction using the 7500 RT-PCR Applied Biosystems system. The difference between the sample gene and the average from the housekeeping genes, β-actin, glyceraldehydes-3-phosphate dehydrogenase (GAPDH), β-2 microglobulin (β2m), β-glucuronidase (Gusb) and heat shock protein 90 α b1 (Hsp90αb1) (ΔCT value) was obtained and the relative fold change was calculated (2^ΔCT^). The 2^ΔCT^ values for the vaccinated mice were compared to unvaccinated infected mouse stomach tissue and expressed as fold-change compared to unvaccinated and infected samples.

### cDNA preparation and validation RT-PCR

Validation for the gene expression of Lipocalin 2 was carried out on individual cDNA samples from all the groups using the RT² qPCR Primer Assay (Qiagen). The Real-time qPCR (RT-PCR) were run in 96-well plates using the standard amplification conditions described for the 7500 RT-PCR system and 40 ng cDNA, 10 μl 2× Power SYBR green master mix (Applied Biosystems, Foster City, CA), and 1 μl of gene-specific oligonucleotide primers. The reactions were run in duplicate, and β-actin was used as the reference gene in all experiments. The difference between β-actin and the target gene (∆*CT*) was determined, and the relative expression was calculated using the formula 2^∆*CT*^. The values were adjusted so that the mean in the infection control group was set to 1. The negative control (lacking reverse transcriptase) giving the lowest *CT* value was used to determine the detection limit.

### Statistical analysis

Analyses were performed using an unpaired two-tailed t-test with Welch´s correction using GraphPad Prism software version 6.0 (GraphPad Software Inc., San Diego, CA). A p value of < 0.05 was considered significant.

## Results

### Sublingually immunized IFN-γ^-/-^ mice are protected against *H*. *pylori* infection mice

IFN-γ^-/-^ and wild-type mice were immunized with *H*. *pylori* lysate antigens and CT via the SL or IG route and then challenged with *H*. *pylori*. Two and five weeks after the challenge, the gastric bacterial loads of the immunized and unimmunized IFN-γ^-/-^ and wild-type mice were determined.

**Fig 1:**
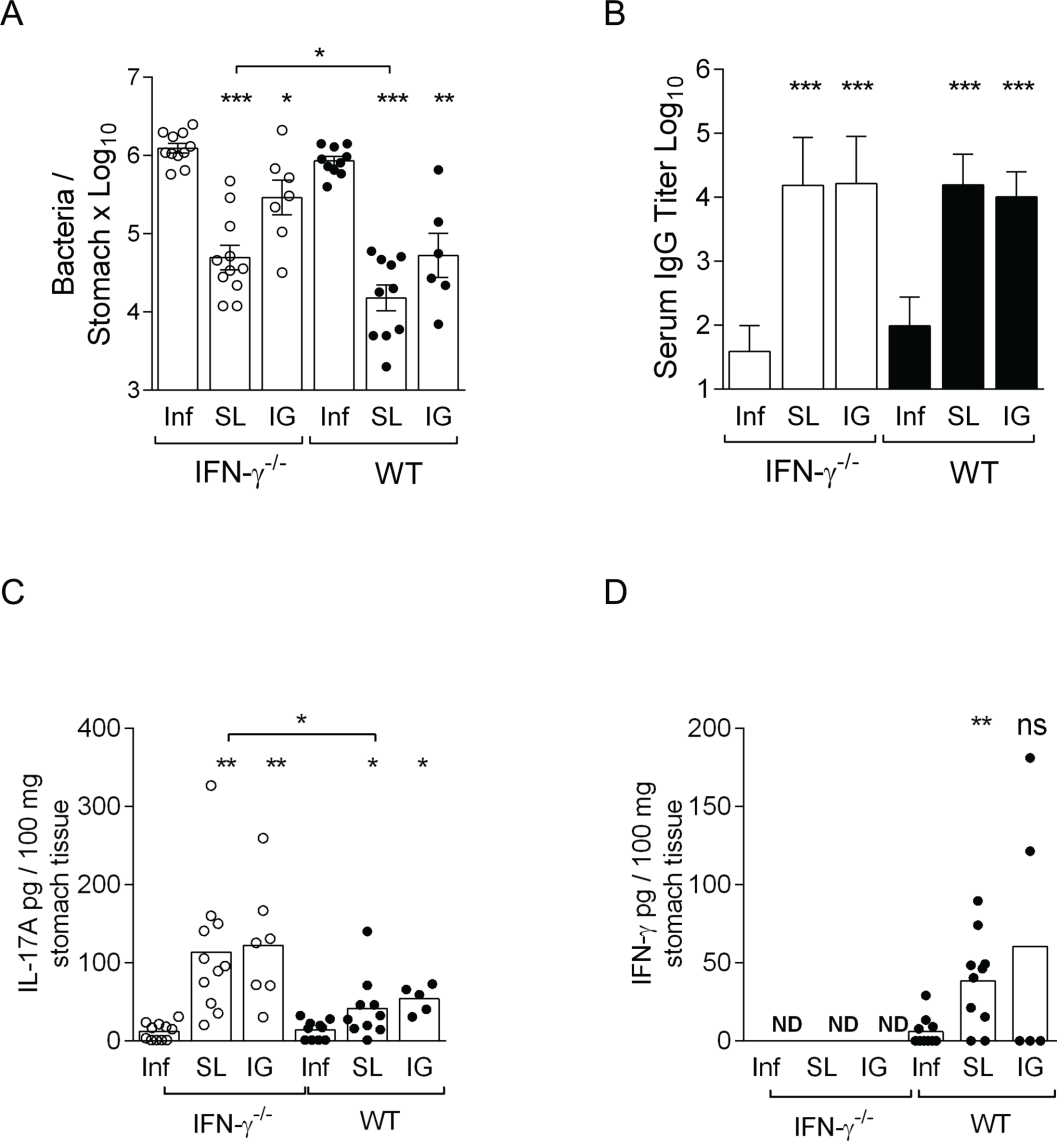
IFN-γ^-/-^ and wild-type mice immunized by the SL or IG route have significantly reduced bacterial counts, generate *H*. *pylori* specific serum IgG titers, and gastric IL-17A responses **.** Groups of IFN-γ^**-/-**^ and wild-type (WT) were immunized via the SL or IG route with *H*. *pylori* lysate antigens and CT (SL or IG) and then infected with live *H*. *pylori* bacteria. Unimmunized (Naïve) mice infected at the same time point served as controls (Inf). At 2 weeks post infection mice were sacrificed and stomach tissue was sampled to determine the level of *H*. *pylori* colonization and gastric tissue cytokine levels. **A.** mean cfu/g stomach for immunized and control WT and IFN-γ^**-/-**^ mice **B.** Mean serum antibody titers of *H*. *pylori* specific antibodies. **C.** IL-17A levels and **D.** IFN-γ levels in stomach tissue extracts in pg/100 mg tissue. Data represent pool of two independent experiments of four with similar results, bars represent mean values of n = 6–11 mice/group,. Statistical analysis was carried out using the unpaired two-tailed t-test with Welch’s correction, denoted by * (p<0.05), ** (p<0.01), *** (p<0.001). ND: Not Detected, ns: not significant.

IFN-γ^-/-^ and wild-type mice immunized by the SL route had significant (25-fold and 56-fold, respectively, p < 0.001) reductions in their *H*. *pylori* bacterial loads compared with unimmunized infected controls (Fig 1A). At 5 weeks post-challenge, the bacterial loads continued to remain significantly lower in sublingually immunized IFN-γ^-/-^ and wild-type mice (138-fold and 286-fold respectively, p < 0.001) compared with unimmunized mice (*data not shown*).

The intragastrically immunized IFN-γ^-/-^ mice and wild-type mice had smaller although significant reductions in their *H*. *pylori* bacterial loads (4-fold, p < 0.05; and 16-fold, p < 0.01, respectively) compared with unimmunized infected mice (Fig 1A). The IG route of immunization tended to be less effective in reducing bacterial loads in the stomach of mice than the SL route for both IFN-γ^-/-^ and wild-type mice.

In general, the mean reduction in the bacterial load after SL or IG immunization was partly affected by the lack of IFN-γ as immunized IFN-γ^-/-^ mice had slightly higher gastric bacterial loads compared with immunized wild-type mice, that was statistically significant in the case of sublingually (p<0.05) but not intragastrically immunized mice (Fig 1A).

The bacterial load was not related to the serum antibody titres in the immunized mice as both IFN-γ^-/-^ and wild-type mice had comparable and significant increases in their serum IgG antibody responses after both SL and IG immunization and challenge (p < 0.001 and p < 0.001) compared with unimmunized infected mice (Fig 1B). In summary, wild- type and IFN-γ^-/-^ mice had significantly reduced bacterial loads after vaccination. Based on fold-change in bacterial load, the SL immunization was more effective than IG immunization in both IFN-γ^-/-^ and wild-type mice.

### Elevated gastric IL-17A responses in the stomach of immunized IFN-γ^-/-^ mice compared to immunized wild-type mice is associated with a lower gastritis score

To correlate the local immune responses to the gastritis and protection in the vaccinated mice, we studied the gastric IFN-γ and IL-17A responses of IFN-γ^-/-^ and wild-type mice. Sublingually immunized IFN-γ^-/-^ mice showed significantly increased levels of IL-17A in the stomach tissue extracts compared with immunized wild-type mice (3-fold, p < 0.05) (Fig 1C). Intragastrically immunized IFN-γ^-/-^ mice also had increased IL-17A levels in the stomach compared with immunized wild-type mice, although this difference was not statistically significant (2-fold, p = ns) (Fig 1C). IFN-γ was undetectable in the stomachs of IFN-γ^-/-^ mice, irrespective of the treatment (*data not shown*). In the stomachs of wild-type mice, IFN-γ levels were elevated in sublingually (3-fold, p< 0.01) and intragastrically (5-fold, p = ns) immunized mice compared with the unimmunized infected controls (Fig 1D).

**Fig 2:**
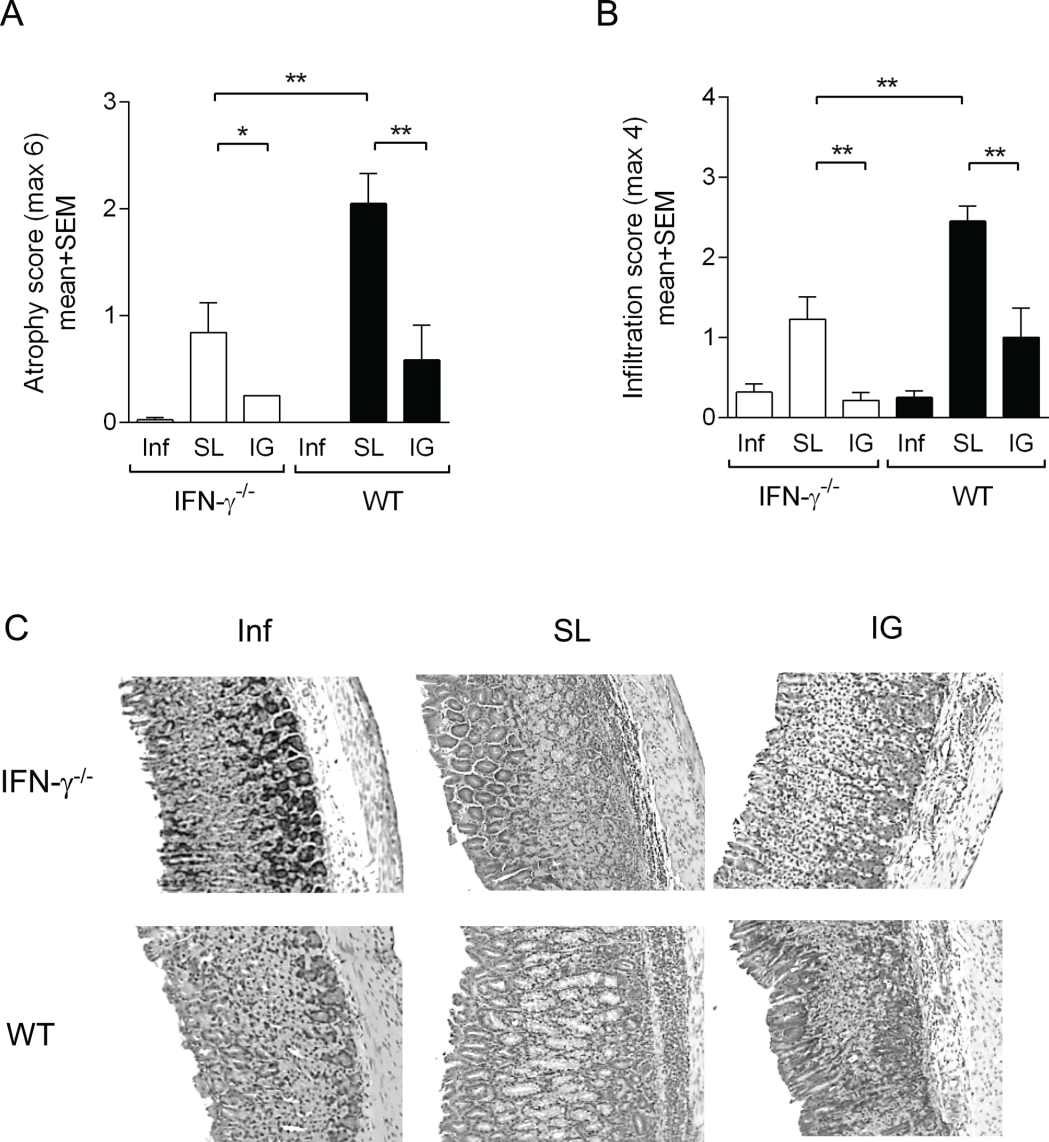
Intragastrically or sublingually immunized IFN-γ^-/-^ and wild-type mice have higher Inflammation and atrophy scores. Groups of IFN-γ^**-/-**^ and wild-type (WT) were immunized via the SL or IG route with *H*. *pylori* lysate antigens and CT (SL or IG) and infected with live *H*. *pylori* bacteria. Unimmunized (Naïve) mice infected at the same time point served as infected controls (Inf). At 2 weeks post-infection the mice were sacrificed. Stomach tissue was sampled to determine the score the inflammation. **A.** Atrophy and **B.** Infiltration scores of formalin fixed stomach tissue stained with hematoxylin and eosin. Data represent pool of two independent experiments of three with similar results, bars represent mean values of n = 6–11 mice/group. Gastritis scores from immunized mice were compared to their respective infected controls and was assessed with the unpaired two-tailed t-test with Welch’s correction and denoted by * (p<0.05), ** (p<0.01), *** (p<0.001). **C.** Microscopy images (100x) of hematoxylin and eosin stained sections of a representative corpus area of stomach from unimmunized infected (Inf), SL and IG immunized IFNγ^**-/-**^ mice (top row) and wild-type mice (bottom row).

We next evaluated the extent of post immunization gastritis in immunized IFN-γ^-/-^ and wild-type mice by grading the atrophy (destruction of parietal and chief cells) and infiltration of immune cells in the corpus area of the stomach tissue. The gastric atrophy and infiltration in the IFN-γ^-/-^ and wild-type unimmunized infection controls were low and comparable between the two groups (Fig 2A and 2B). When compared with their respective unimmunized infection controls, sublingually immunized IFN-γ^-/-^ and wild-type mice had significantly higher atrophy scores (p < 0.01 and p < 0.001 respectively) (Fig 2A and 2C). Similar results were observed when grading the tissue for infiltration in sublingually immunized IFN-γ^-/-^ mice (p < 0.05) and immunized wild-type mice (p < 0.001) when compared with their respective unimmunized infection controls (Fig 2B and 2C). However, sublingually immunized IFN-γ^-/-^ mice had significantly lower atrophy and infiltration (p < 0.01) compared with sublingually immunized wild-type mice (Fig 2A, 2B and 2C). Finally, intragastrically immunized IFN-γ^-/-^ and wild type mice had significantly lower atrophy and infiltration scores in the stomach post-challenge compared to sublingually immunized IFN-γ^-/-^ and wild type (Fig 2A, 2B and 2C). We found no significant difference in the gastritis scores between intragastrically immunized IFN-γ^-/-^ and wild type mice. In summary, the elevated IL-17A responses in the stomach of sublingually immunized IFN-γ^-/-^ mice had minor influence on the gastritis (atrophy and infiltration) scores compared to sublingually immunized wild-type mice.

### In vitro proliferative response to *H*. *pylori* antigens and IL-17A secretion is intact in immunized IFNγ^-/-^ mice

**Fig 3:**
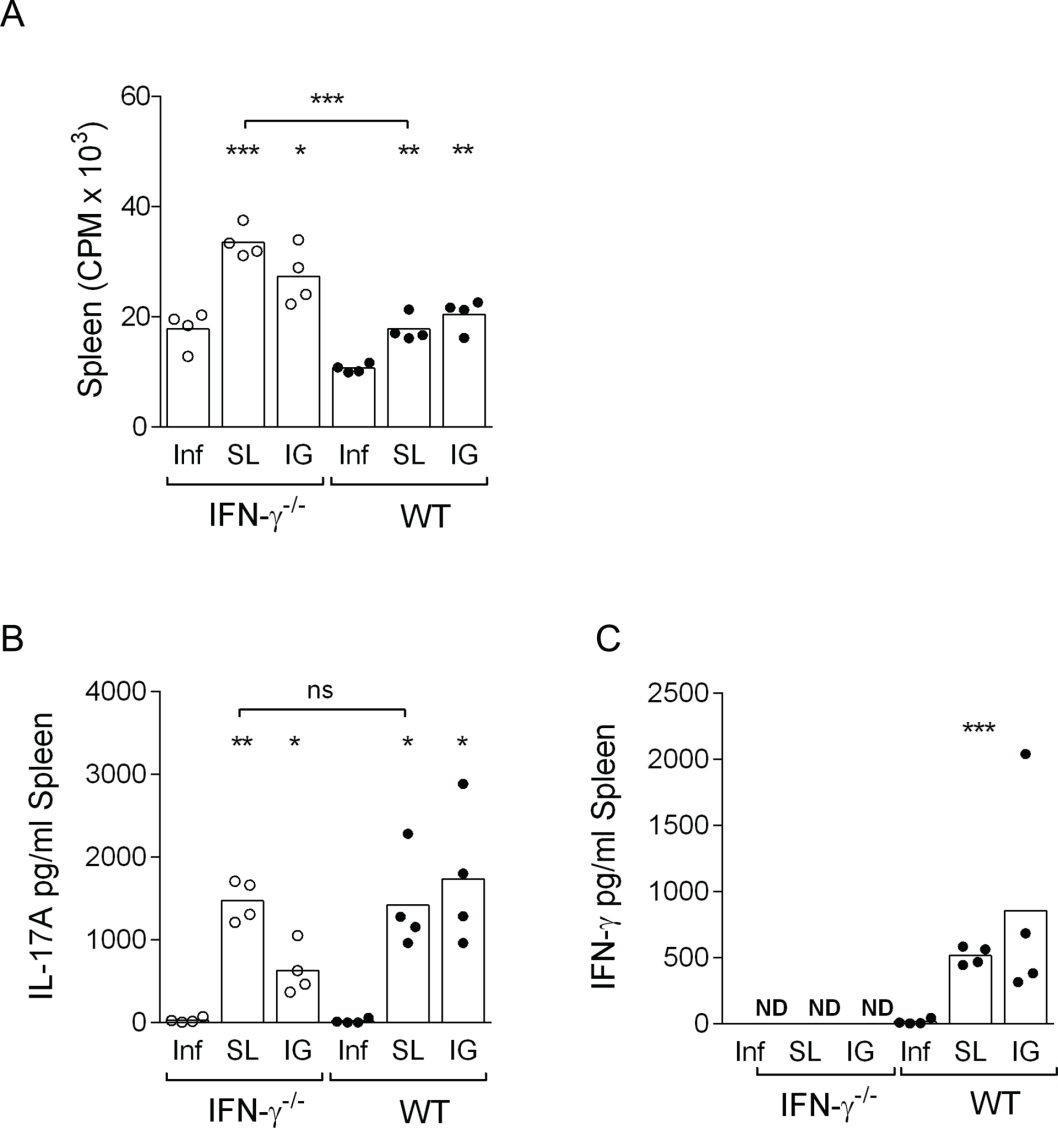
Splenocytes from both intragastrically and sublingually immunized IFN-γ^-/-^ mice and wild-type mice have increased *in vitro* proliferative responses, and cytokine secretion. Groups of IFNγ^**-/-**^ and wild-type (WT) mice were sublingually (SL) or intragastrically (IG) immunized with *H*. *pylori* lysate antigens and CT or left unimmunized (Inf) and then challenged with live *H*. *pylori* bacteria. Mice were sacrificed and spleen cells were isolated **A.** Single cell suspensions were prepared and cultured in 6 replicate wells together with *H*. *pylori* lysate antigens. Counts per minute (cpm) of incorporated radioactive thymidine were used as a measure of proliferation of the cells. Each dot represents an individual mouse and the bars the mean of the group. Representative data of one of three independent experiments. Supernatants were collected from *in vitro* cultured spleen cells (from A) and assessed for cytokines **B.** IL-17A and **C.** IFN-γ. Values are shown in pg/ml. Levels in immunized mice were compared to their respective infection controls and was assessed with the unpaired two-tailed t-test with Welch’s correction and denoted by * (p<0.05), ** (p<0.01), *** (p<0.001). ND: Not Detected, ns: not significant

To study the possible effects of lack of IFN-γ on CD4+ T cell priming and Th1 and Th17 polarization we prepared single-cell suspensions of spleen cells from sublingually or intragastrically immunized IFN-γ^-/-^ and wild-type mice and also unimmunized infected mice. Cells were cultured *in vitro* in the presence of *H*. *pylori* lysate antigens, and the proliferative response and cytokine secretion analyzed. There was a significant difference in the capacity of the cells from immunized IFN-γ^-/-^ or wild-type mice to respond to *H*. *pylori* antigens compared to unimmunized infection controls (Fig 3A). There was even a tendency for higher proliferative response from cells isolated from immunized IFNγ^-/-^ compared to WT mice which was significant in sublingually immunized mice (p<0.01). The IL-17A and IFN-γ secretion in the culture supernatant from spleen cells was analyzed and found to be elevated in immunized compared to unimmunized infection controls (Fig 3B and 3C). The secretion of IL-17A from cells isolated from immunized IFN-γ^-/-^ and wild-type mice was comparable (Fig 3B). In summary, our results demonstrate that irrespective of the immunization route (SL or IG) the vaccine-induced proliferative and IL-17A responses to *H*. *pylori* antigens are intact in the absence of IFNγ.

### The CD3^+^CD4^+^ T cells are source of IL-17A production in the spleen and MLN of immunized IFN-γ^-/-^ and wild-type mice

**Fig 4:**
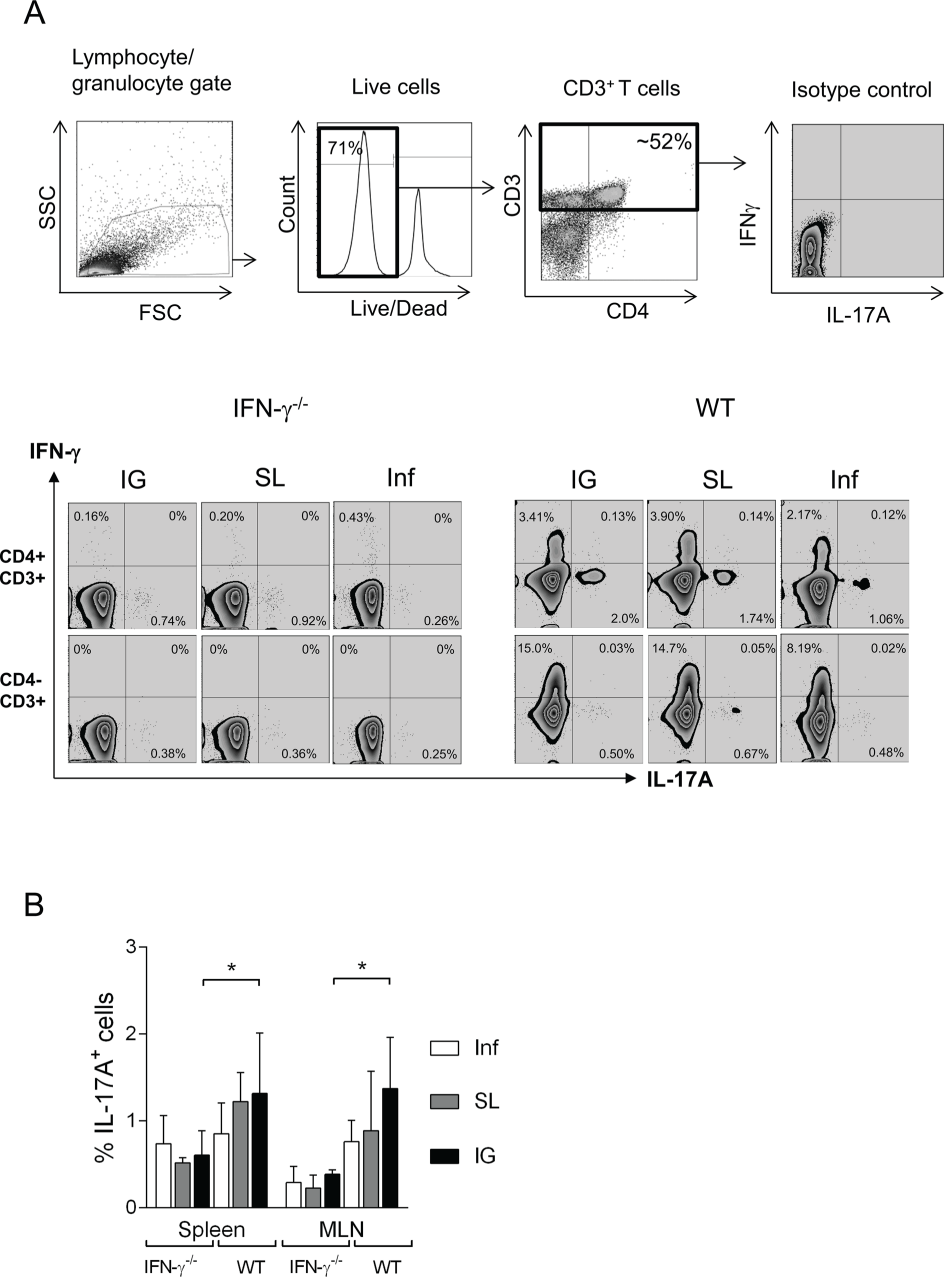
IL-17A and IFN-γ production from Spleen and MLN cells of intragastrically or sublingually immunized IFN-γ^-/-^ and wild-type mice. Groups of IFN-γ^**-/-**^ and wild-type (WT) were immunized via the SL or IG route with *H*. *pylori* lysate antigens and CT (SL or IG) or left unimmunized (Inf) and then challenged with live *H*. *pylori* bacteria. At three weeks post challenge mice were sacrificed and spleen and MLN was taken for flow cytometric analysis. Spleen and MLN was taken from individual mice (n = 4) and single cell single cell suspensions were prepared and stimulated *in vitro* with PMA and Ionomycin and then stained for further flow cytometric analysis. **A** Gating strategy showing analysis of IL-17A and IFN-γ secreting cells among live CD3+ T cells. **B** Bar graph shows frequency mean + SD of IL-17A^**+**^ cells in spleen and MLN. Data represent one of two independent experiments.

We next addressed by flow cytometry, the source of IL-17A secretion by spleen and MLN cells isolated from immunized or unimmunized mice 3 weeks post-challenge. Cell suspensions were prepared and stimulated *in vitro* and subsequently stained for cell surface markers and intracellular IL-17A and IFN-γ. There was no significant difference in the proportion of IL-17A secreting cells detected in either the spleen or the MLN in either IFN-γ^-/-^ or wild-type mice, except that IG vaccination appeared to induce higher frequency of IL-17A^+^ cells in both organs (Fig 4A and 4B). The primary producers of IL-17A in infected or immunized mice were CD3^+^CD4^+^ cells, while the source of IFN-γ in immunized wild-type mice post-infection was the CD3^+^CD4^-^ cells and to a lesser extent by the CD3^+^CD4^+^ population (Fig 4A and *data not shown*). In summary, we have identified the CD3^+^CD4^+^ cells to be main population of cells secreting IL-17A in the spleen and MLN during *H*. *pylori* infection, while CD3^+^CD4^-^ cells to be the main producers of IFN-γ.

### Neutralization of IL-17A in sublingually immunized IFN-γ^-/-^ mice abrogated the protection, anti-microbial response and proliferation by MLN cells in response to *H*. *pylori* antigens

**Fig 5:**
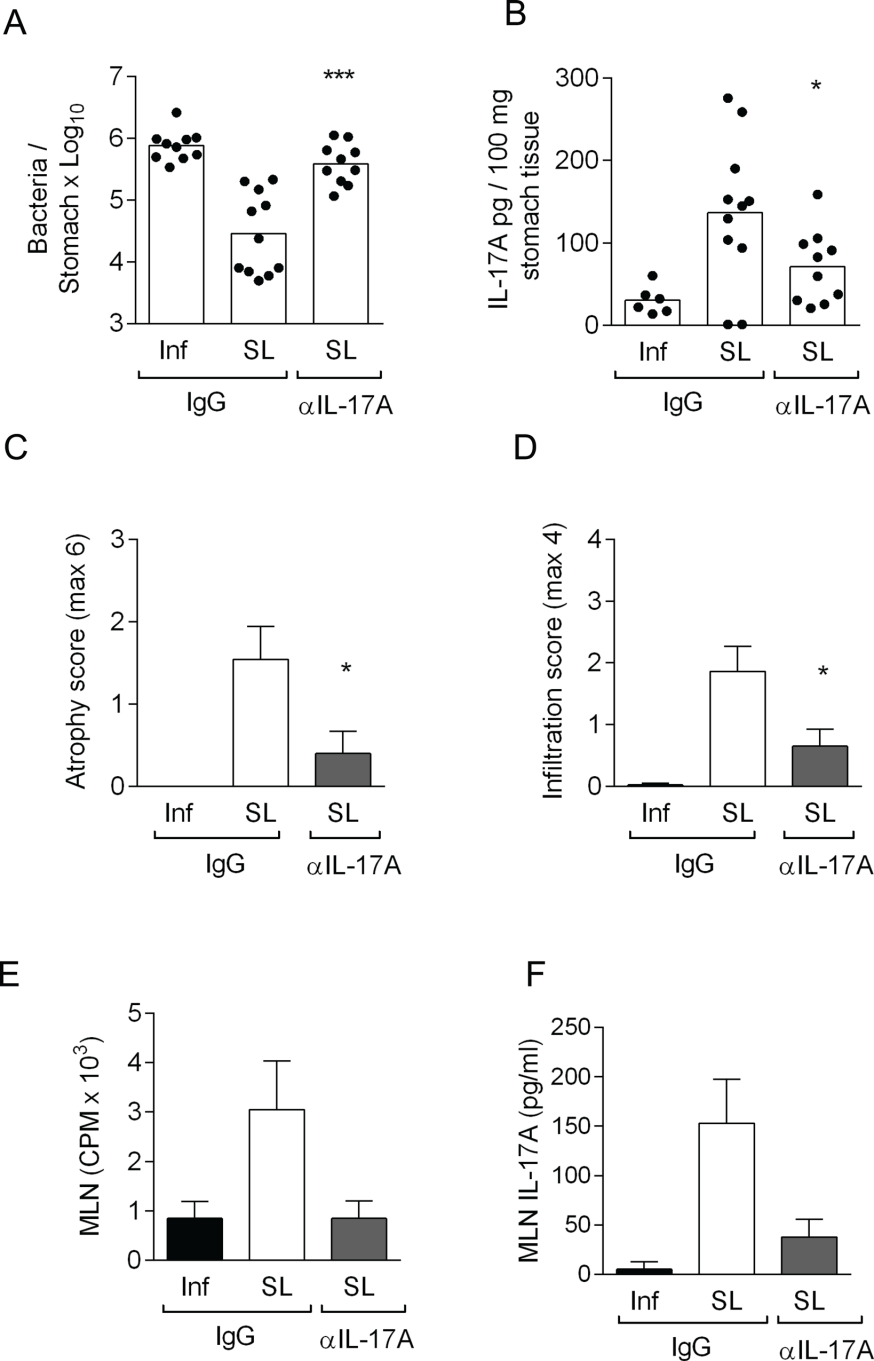
Neutralization of IL-17A abrogates protection, and reduces gastric inflammation and proliferation of MLN cells in sublingually immunized IFN-γ^-/-^ mice. IFN-γ^**-/-**^ mice were sublingually immunized with *H*. *pylori* lysate antigens and CT (SL) or left unimmunized (Inf) and infected with live *H*. *pylori* bacteria. Mice were injected intraperitoneally neutralizing IL-17A antibody (αIL-17A) or control IgG antibody (IgG). Two weeks post infection mice were sacrificed. **A.** Stomach tissue was analyzed for *H*. *pylori* colonization by quantitative culture and expressed as mean log_10_ cfu per stomach, and SEM. **B.** analysis of IL-17A secretion in stomach tissue extracts and **C.** Atrophy and **D.** Infiltration in stomach tissue was scored. n = 6–11 mice/group, pool of two experiments. Bars represent mean. Statistically significant difference between sublingually immunized IFN-γ^**-/-**^ mice injected neutralizing IL-17A antibody compared to immunized mice injected isotype control antibody was calculated by an unpaired two-tailed t-test with Welch correction and denoted by * (p<0.05), ** (p<0.01), *** (p<0.001). **F.** single cell suspensions of MLN were prepared and cultured *in vitro* with *H*. *pylori* lysate antigens. Counts per minute (cpm) of incorporated radioactive thymidine was used as a measure of proliferation of the cells. Bars represent mean value and standard deviation (SD) counts in 6 individual wells in pooled mice (n = 5–7 mice/group) **G.** Supernatants were collected from *in vitro* cultured MLN (from D) and assessed for IL-17A shown in pg/ml, of six pooled wells. Data pool of two independent experiments. **E.** Stomach tissue was analyzed for gene expression of Lcn (Lipocalin-2) and expressed as relative gene expression where unimmunized infection control was set to 1. Statistically significant difference between sublingually immunized IFN-γ^**-/-**^ mice injected neutralizing IL-17A antibody compared to immunized mice injected isotype control antibody was calculated by an unpaired two-tailed t-test with Welch’s correction and denoted by *** (p<0.001).

Since our results until now suggested that bacterial reduction can be induced in IFN-γ^-/-^ mice after immunization with enhanced IL-17A secretion, we were therefore interested in studying the effects of removal of IL-17A during the effector phase, after sublingual immunization and infection in IFN-γ^-/-^ mice. We report that, neutralization of IL-17A in sublingually immunized IFN-γ^-/-^ mice post-infection resulted in a significant (p < 0.001) increase in the bacterial load compared with sublingually immunized mice injected the isotype control IgG antibody (Fig 5A). Stomach tissue extracts were further analyzed to evaluate the efficiency of *in vivo* neutralization of IL-17A. A two-fold reduction in IL-17A protein levels was observed in the stomachs of immunized mice receiving neutralizing IL-17A antibody compared with mice administered the isotype control IgG antibody (Fig 5B). The effect of IL-17A neutralization on the gastritis (atrophy and infiltration) in sublingually immunized IFN-γ^-/-^ mice was also evaluated and found to be significantly lower than that in sublingually immunized IFN-γ^-/-^ mice receiving the isotype control IgG antibody (Fig 5C and 5D).

To investigate the effect of neutralizing IL-17A on cellular immune responses, single cell suspensions from MLN were prepared and stimulated *in vitro* with *H*. *pylori* antigens. MLN cells from sublingually immunized mice administered an isotype control IgG antibody showed enhanced proliferation to *H*. *pylori* antigens compared with those from unimmunized infected mice (Fig 5E). Remarkably, in sublingually immunized IFN-γ^-/-^ mice receiving neutralizing antibodies to IL-17A, the MLN cell proliferation was reduced to the level of unimmunized infected mice administered isotype control IgG antibody (Fig 5E). MLN cells were also evaluated for their secretion of cytokines in cell culture supernatants. An impaired secretion of IL-17A in sublingually immunized mice receiving neutralizing IL-17A compared with sublingually immunized mice receiving the isotype control was observed (Fig 5F).

To further understand the mechanisms whereby sublingually immunized IFN-γ^-/-^ mice could control their bacterial colonization and the role of IL-17A in this process, we carried out real-time PCR array (antibacterial response) analysis of the stomach tissue from IFN-γ^-/-^ mice administered anti-IL-17A antibody or isotype control. Reduced expression of antimicrobial peptide related genes, *Camp* (Cathelicidin), *Lcn2* (Lipocalin 2) and *Ltf* (Lactotransferrin) was seen in the stomach tissue of sublingually immunized IFN-γ^-/-^ mice administered anti-IL-17A compared to immunized mice administered isotype control antibody (Table 1A). In addition, cytokine *IL-6*, chemokines (*Cxcl1*, *Cxcl3*) and inflammasome pathway related genes (*Casp1* and *Nlrp1*) were less expressed in stomach tissue after IL-17A neutralization compared to mice administered isotype control antibody after sublingual vaccination (Table 1B and 1C). Finally, validation PCRs in individual mice showed that *Lcn2* expression was significantly lower in the stomach of immunized mice administered anti-IL-17A compared to immunized mice administered isotype control antibody (Fig 5G) with a tendency also for lower expression of *Ltf* and *Defb3* (mouse β-defensin-3) (*data not shown*).

**Table 1:** Relative Gene expression in stomach tissue after SL vaccination and IL-17A neutralization compared to non-immunized controls.

**A Antimicrobial peptides**		
	*H*. *pylori* Lysate + CT	
Gene	IgG	αIL-17A
*Camp* (Cathelicidin)	4.83	2.60
*Ctsg* (Cathepsin G)	0.37	0.35
*Lcn2* (Lipocalin 2)	5.43	0.53
*Lcf* (Lactotransferrin)	9.16	2.01
*Lyz2* (Lysozyme 2)	0.86	0.52
*Mpo* (Myeloperoxidase)	1.47	0.39
*Prtn3* (Proteinase 3)	1.06	0.38
*Slpi* (Secretory leukocyte peptidase inhibitor)	1.52	1.56
**B Cytokines and Chemokines**		
	*H*. *pylori* Lysate + CT	
Gene	IgG	αIL-17A
*Ccl3* (MIP-1α)	0.50	0.54
*Ccl4* (MIP-1β)	1.52	1.05
*Ccl5* (RANTES)	0.83	0.66
*Cxcl1* (Groα/MIP-2/KC)	3.88	0.61
*Cxcl3* (Groα/MIP-2/KC)	2.43	0.86
*Il12a*	0.95	0.75
*Il12b*	0.87	0.86
*Il18*	0.66	0.58
*Il1b*	1.80	0.70
*Il6*	4.13	1.50
**C Inflammasome**		
	*H*. *pylori* Lysate + CT	
Gene	IgG	αIL-17A
*Casp1* (Caspase 1)	3.64	1.10
*Naip1* (NAIP-1)	1.94	0.37
*Nlrc4* (NLRC-4)	1.14	0.31
*Nlrp1a* (NALP-1A)	2.18	1.75
*Nlrp3* (NALP-3)	1.51	0.41
*Pycard* (PYD and CARD domain containing)	1.17	0.70

§ RT-PCR array on stomach tissue. The difference between housekeeping gene β-actin and the target gene (∆*CT*) was determined, and the relative expression was calculated using the formula 2^∆*CT*^. Values are then expressed as fold change in gene expression in vaccinated mice compared to stomach tissue taken from unvaccinated infected mice.

Taken together, the *in vivo* administration of neutralizing IL-17A antibody after SL immunization and challenge in IFN-γ^-/-^ mice resulted in (i) abolished protection against *H*. *pylori* infection, (ii) dampened gastric inflammation, (iii) reduced expression of antimicrobial response genes and (iv) impaired MLN cell proliferation and IL-17A secretion in response to *H*. *pylori* antigens.

## Discussion

Protection against *H*. *pylori* infection has been shown to be associated with post-immunization gastritis, and an increase in both IFN-γ and IL-17A in the stomachs of immunized mice [17]. The aim of the current study was to determine whether protection could be separated from post-immunization gastritis after SL immunization by studying the role of IFN-γ and IL-17A in these two processes. We report that the protection against *H*. *pylori* infection after SL with *H*. *pylori* lysate antigens and cholera toxin can be induced in the absence of IFN-γ although to somewhat lower levels compared to immunized WT mice. Furthermore, the sublingually immunized IFN-γ^-/-^ mice had significantly elevated IL-17A levels in the stomach tissue compared with immunized wild-type mice. Indeed, *in vivo* neutralization of IL-17A in sublingually immunized IFN-γ^-/-^ mice abrogated the protection and anti-microbial response against *H*. *pylori* infection.

Different mucosal routes of immunization have rarely been compared in the same study when evaluating candidate vaccine preparations against *H*. *pylori* infection. One could hypothesise that differences in the environment and the stability of the antigens and adjuvant at the two sites (SL/IN versus IG) may contribute to differences in the induction of immune responses and, consequently, protection against *H*. *pylori* infection. Indeed, we have demonstrated using the same dose of the vaccine, that in mice immunized with recombinant HpaA and UreB and CT [29] or *H*. *pylori* lysate antigens and dmLT [25], the protection against *H*. *pylori* was significantly better in sublingually than in intragastrically immunized mice. Furthermore, we have preliminary data showing a higher frequency of IFN-γ^+^ and IL-17A^+^ cells in the draining cervical lymph nodes after SL immunization with *H*. *pylori* antigens and CT than in the MLN after IG immunization (*Sjökvist-Ottsjö et al*. *manuscript in preparation*), suggesting that, when using CT as an adjuvant, the route of immunization may affect T cell priming and cytokine production and subsequently protection against *H*. *pylori* infection. Other possibilities that require further investigation when immunizing via the intragastric route include the dose, timing and number of immunizations, which may also have an influence on the immune response and protection against *H*. *pylori* [30].

In the stomachs of sublingually immunized IFN-γ^-/-^ mice, the IL-17A levels were elevated with significantly lower atrophy and infiltration scores compared with sublingually immunized wild-type mice. In agreement with our results, a recent study by Ding et al. demonstrated that intranasally immunized IL-12p35^-/-^ mice (deficient in IFN-γ responses) were as well protected against *H*. *pylori* infection as wild-type mice, and showed increased gastric IL-17A gene expression in the stomach [31]. These data suggest that in the absence of inhibitory signals from Th1 cytokines, IL-17A responses dominate [32] and contribute to the reduction in the gastric bacterial loads of immunized mice. We also observed elevated IL-17A in the stomach tissue of intragastrically immunized IFN-γ^-/-^ mice and that these mice were less protected against *H*. *pylori* infection compared with sublingually immunized IFN-γ^-/-^ mice. One key difference in the gastric immune response between the intragastrically and sublingually immunized IFN-γ^-/-^ mice was the inflammation score. The sublingually immunized IFN-γ^-/-^ mice had a significantly higher inflammation scores than intragastrically immunized IFN-γ^-/-^ mice. Thus, our data suggests that the mechanism by which mice are protected against *H*. *pylori* infection involves IL-17A and additional as-yet-unknown accessory cells that are more effectively recruited to the stomach by SL than IG immunization. Therefore, future studies should address in detail the phenotype of the immune cell populations that migrate into the stomachs of sublingually or intragastrically immunized IFN-γ^-/-^ mice and secrete IL-17A in response to *H*. *pylori* infection.

Previous studies in wild-type mice have shown that the vaccine-induced protection against *H*. *pylori* infection was associated with post-immunization gastritis [5, 9, 33, 34]. And there has been one report of post-immunization gastritis, in human volunteers that were vaccinated against *H*. *pylori* and then challenged [35]. The gastritis in mice is characterized by the infiltration of CD4^+^ T cells, B cells, neutrophils, mast cells and eosinophils that secrete cytokines or mediators, presumably making the environment unfavourable for the colonization of *H*. *pylori* [5, 8]. Sublingually immunized IFN-γ^-/-^ mice were able to significantly reduce colonization, but also had significantly decreased the inflammation scores compared to immunized wild type mice. In keeping with our observations, liposome-mediated depletion of macrophages has been shown to reduce the gastric pathology of *H*. *pylori*-infected mice, but did not affect the protection [36]. Further, the recent study by Ding et al has shown that the treatment of *Helicobacter*-infected mice with recombinant IL-12 (which induces IFN-γ production by T cells) results in enhanced gastritis and reduced bacterial loads compared with untreated mice [31]. Unfortunately, the frequency or activation of macrophages was not addressed in that study. Our data suggest that the elevated levels of gastric IFN-γ in *H*. *pylori*-infected and vaccinated mice is pro-inflammatory, potentially through the activation of M1 macrophages we have previously shown to be accumulated in the stomach of vaccinated mice post-infection [37].

Our observation that the neutralization of IL-17A in immunized IFN-γ^-/-^ mice abrogated the protection against *H*. *pylori* infection is consistent with previous reports on the role of IL-17A in reducing bacterial load in the stomach of mice [17, 18]. We further report that MLN cells, isolated from sublingually immunized IFN-γ^-/-^ mice injected with IL-17A neutralizing antibody, showed a dampened *in vitro* proliferative response to *H*. *pylori* antigens. These findings suggest that whereas SL immunization likely expands a pool of *H*. *pylori*-specific T cells that secrete IL-17A, the challenge further expands this pool of IL-17A-secreting cells (which was blocked by the neutralizing antibody), followed by their migration to the stomachs of infected mice and the consequent reduction in bacterial numbers. Thus, while IL-17A seems to be dispensable during the priming phase of the immune response, it is critical during the effector phase through effect on neighbouring cells (see below) and can remarkably reduce the bacterial load in the stomach of vaccinated mice with minimal inflammation.

IL-17A has been shown to have a protective role in other mucosal infections including *Citrobacter rodentium*, an extracellular Gram-negative gut bacterium that naturally infects mouse colonies. The generation of protective Th17 responses in *C*. *rodentium* infection had been found to be IL-23 dependent. Additionally, IL-22 may contribute to early host defence through the induction of antimicrobial peptides in colonic epithelial cells. As proposed by several reports and now also supported by our results, Th17 cytokines may have a protective function for the effective host defence against extracellular pathogens at mucosal sites [38, 39]. These results are not surprising, considering some of the known functions of IL-17A: the secretion of IgA, recruitment of neutrophils and upregulation of anti-microbial peptides by epithelial cells [40].

Indeed, we observed that IL-17A neutralization in sublingually immunized IFN-γ^-/-^ mice led to a decrease in gene expression of cathelicidin, lactotransferrin and lipocalin 2, suggesting that anti-microbial peptides and iron chelating proteins maybe an important component of the local anti-microbial response in against *H*. *pylori* infection affected by IL-17A. In keeping with these results, expression of *Lcn2* was also increased compared to unimmunised controls in mice immunised using a recombinant attenuated salmonella-based *H*. *pylori* vaccine [39]. Several studies have also shown an inhibitory effect of antimicrobial peptides, lactotransferrin and lipocalin-2 on *H*. *pylori* growth in vitro [41–43]. Our data suggests that the lack of protection seen after IL-17A neutralization in IFN-γ^-/-^ mice is partly due to a dampened antimicrobial response.

In conclusion, we report protection against *H*. *pylori* infection in IFN-γ^-/-^ mice after SL and IG immunization, although to a lesser extent than wild-type mice. The reduction in the gastric bacterial loads of sublingually immunized IFN-γ^-/-^ mice was associated with elevated IL-17A responses, and reduced post-immunization gastritis compared with sublingually immunized wild-type mice. The neutralization of IL-17A in IFN-γ^-/-^ mice abolished the protection against *H*. *pylori* infection. Our results suggest that the post-immunization gastritis of sublingually immunized mice may be promoted by CD3^+^CD4^-^ derived IFN-γ (and possibly other pro-inflammatory cytokines), whereas CD3^+^CD4^+^ derived IL-17A appears to be specific to the anti-bacterial response to *H*. *pylori* infection. The importance of IL-17A for protection against *H*. *pylori* infection will have implications for the design of anti-*H*. *pylori* vaccines as the composition, formulation and the route of immunization may influence the induction of appropriate immune responses to clear the infection with minimal post-immunization gastritis.

## Supporting Information

S1 Data (ZIP)
