# Correction: Defining the Roles of IFN-γ and IL-17A in Inflammation and Protection against *Helicobacter pylori* Infection

**DOI:** 10.1371/journal.pone.0142747

**Published:** 2015-11-06

**Authors:** Louise Sjökvist Ottsjö, Carl-Fredrik Flach, Staffan Nilsson, Rene de Waal Malefyt, Anna K. Walduck, Sukanya Raghavan

Fig 5 is incorrect, and there are a number of errors in the caption for Fig 5. Please see the corrected Fig 5 and its caption here.

**Fig 5:**
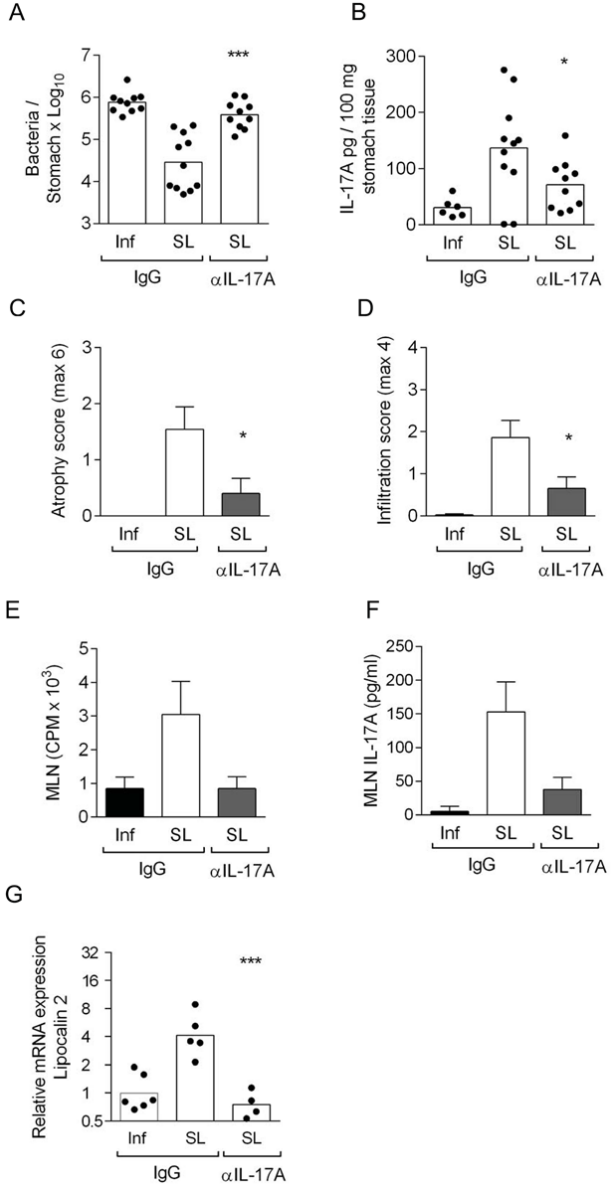
Neutralization of IL-17A abrogates protection, and reduces gastric inflammation and proliferation of MLN cells in sublingually immunized IFN-γ-/- mice. IFN-γ-/- mice were sublingually immunized with *H*. *pylori* lysate antigens and CT (SL) or left unimmunized (Inf) and infected with live *H*. *pylori* bacteria. Mice were injected intraperitoneally neutralizing IL-17A antibody (αIL-17A) or control IgG antibody (IgG). Two weeks post infection mice were sacrificed. **A.** Stomach tissue was analyzed for *H*. *pylori* colonization by quantitative culture and expressed as mean log10 cfu per stomach, and SEM. **B.** analysis of IL-17A secretion in stomach tissue extracts and **C.** Atrophy and **D.** Infiltration in stomach tissue was scored. n = 6–11 mice/group, pool of two experiments. Bars represent mean. Statistically significant difference between sublingually immunized IFN-γ-/- mice injected neutralizing IL-17A antibody compared to immunized mice injected isotype control antibody was calculated by an unpaired two-tailed t-test with Welch correction and denoted by * (p<0.05), ** (p<0.01), *** (p<0.001). **E.** single cell suspensions of MLN were prepared and cultured in vitro with *H*. *pylori* lysate antigens. Counts per minute (cpm) of incorporated radioactive thymidine was used as a measure of proliferation of the cells. Bars represent mean value and standard deviation (SD) counts in 6 individual wells in pooled mice (n = 5–7 mice/group) **F.** Supernatants were collected from in vitro cultured MLN (from E) and assessed for IL-17A shown in pg/ml, of six pooled wells. Data pool of two independent experiments. **G.** Stomach tissue was analyzed for gene expression of Lcn (Lipocalin-2) and expressed as relative gene expression where unimmunized infection control was set to 1. Statistically significant difference between sublingually immunized IFN-γ-/- mice injected neutralizing IL-17A antibody compared to immunized mice injected isotype control antibody was calculated by an unpaired two-tailed t-test with Welch's correction and denoted by *** (p<0.001).
