# Supplementary figures and images for: Defining the Roles of IFN-γ and IL-17A in Inflammation and Protection against Helicobacter pylori Infection
Source: PLoS One. 2015 Jul 13;10(7):e0131444. doi: 10.1371/journal.pone.0131444 (PMC4500503; doi:10.1371/journal.pone.0131444)

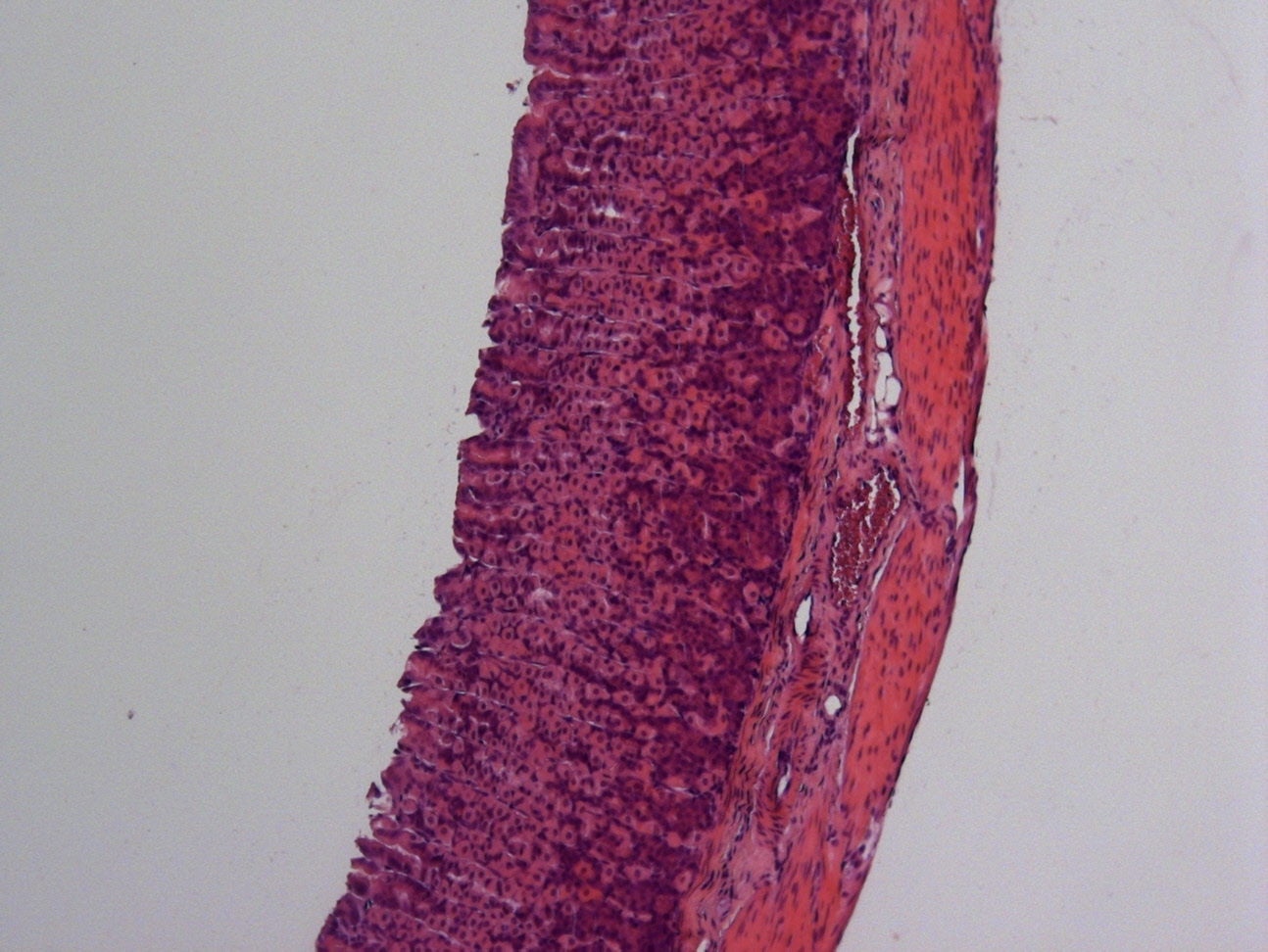

Supplement: S1 Data — (ZIP) [file pone.0131444.s001.zip › Raw data for PONE-D-14-46387R1/IFNg KO IG.JPG]

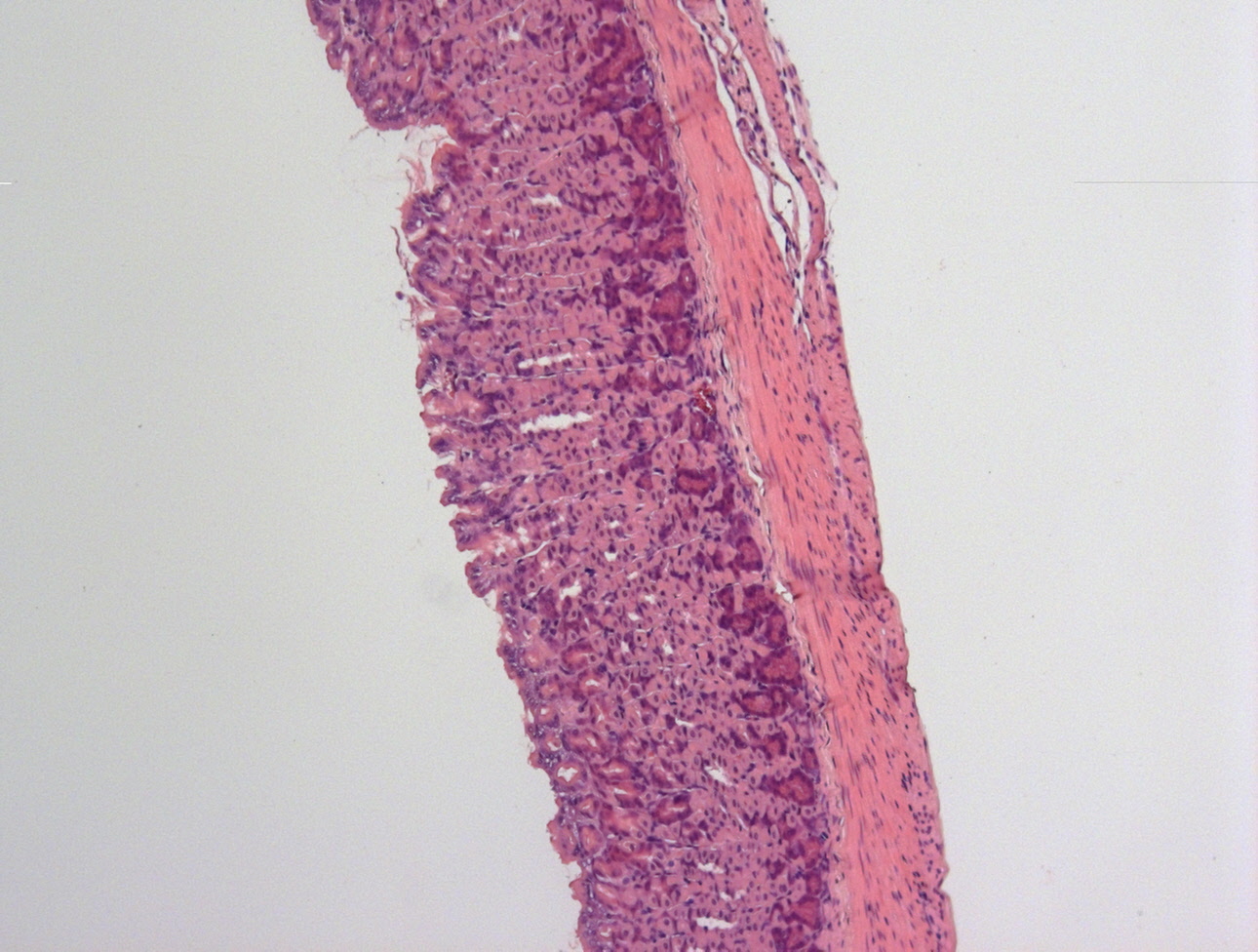

Supplement: S1 Data — (ZIP) [file pone.0131444.s001.zip › Raw data for PONE-D-14-46387R1/IFNgKO INF.JPG]

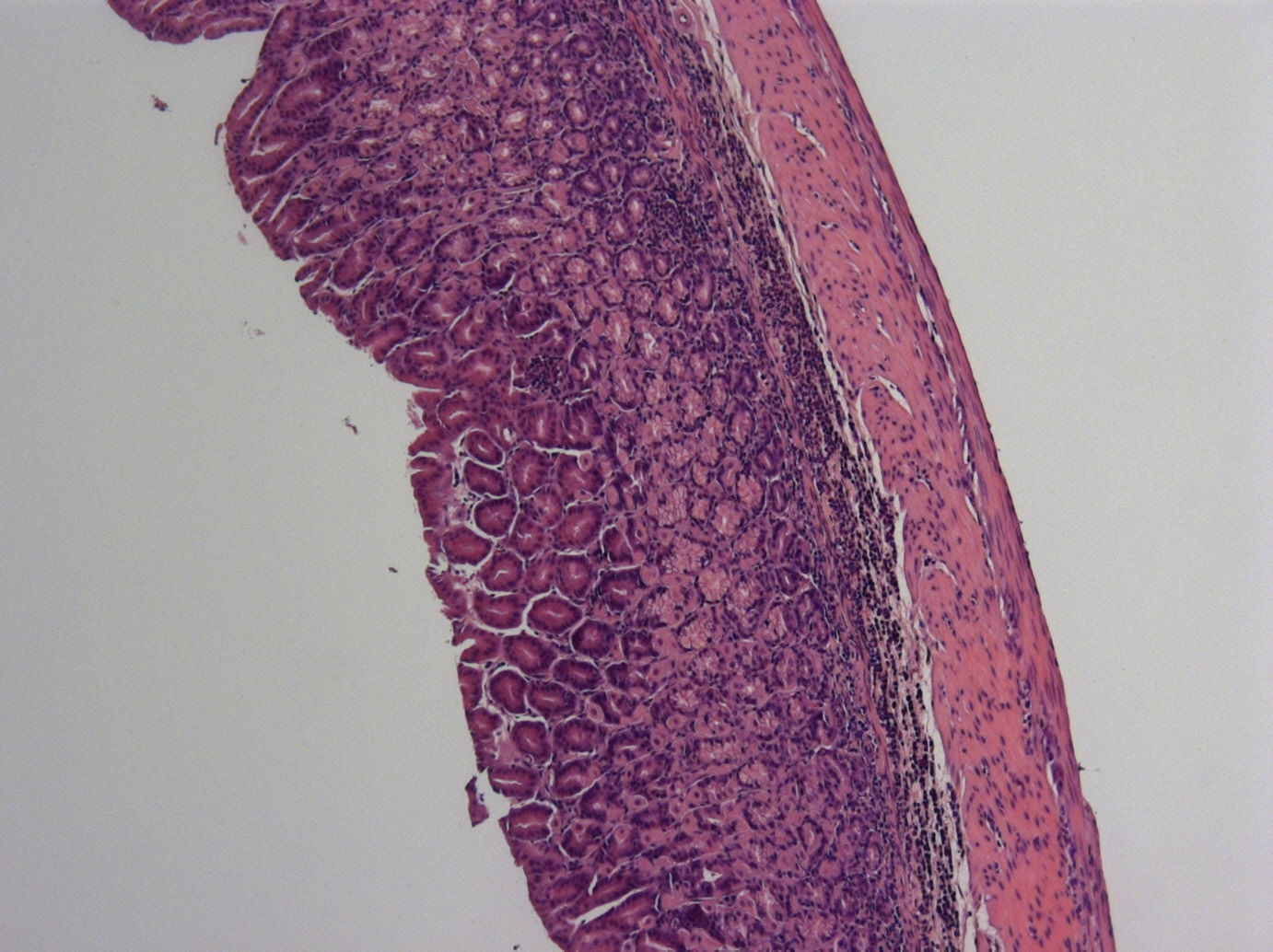

Supplement: S1 Data — (ZIP) [file pone.0131444.s001.zip › Raw data for PONE-D-14-46387R1/IFNgKO SL.JPG]

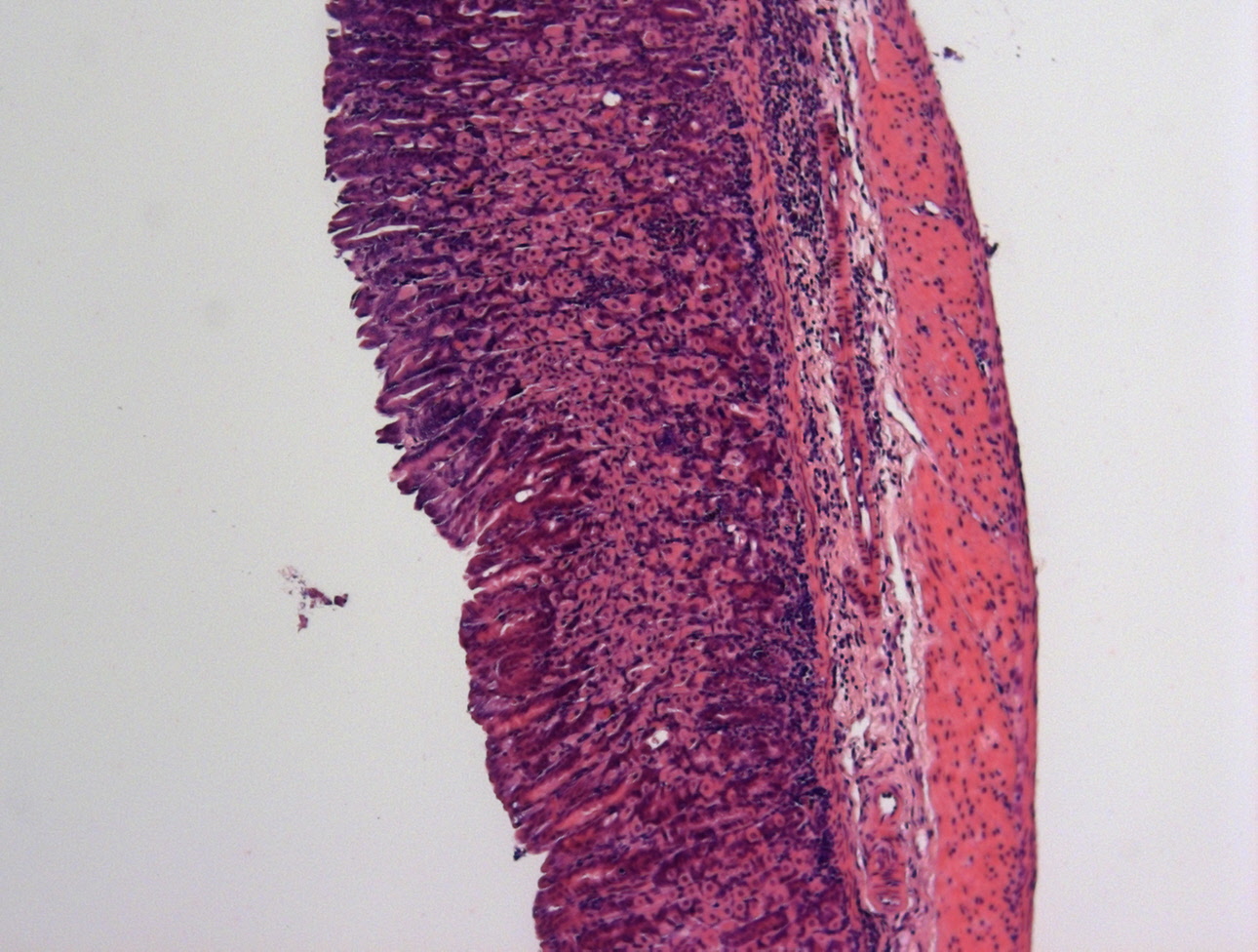

Supplement: S1 Data — (ZIP) [file pone.0131444.s001.zip › Raw data for PONE-D-14-46387R1/WT IG.JPG]

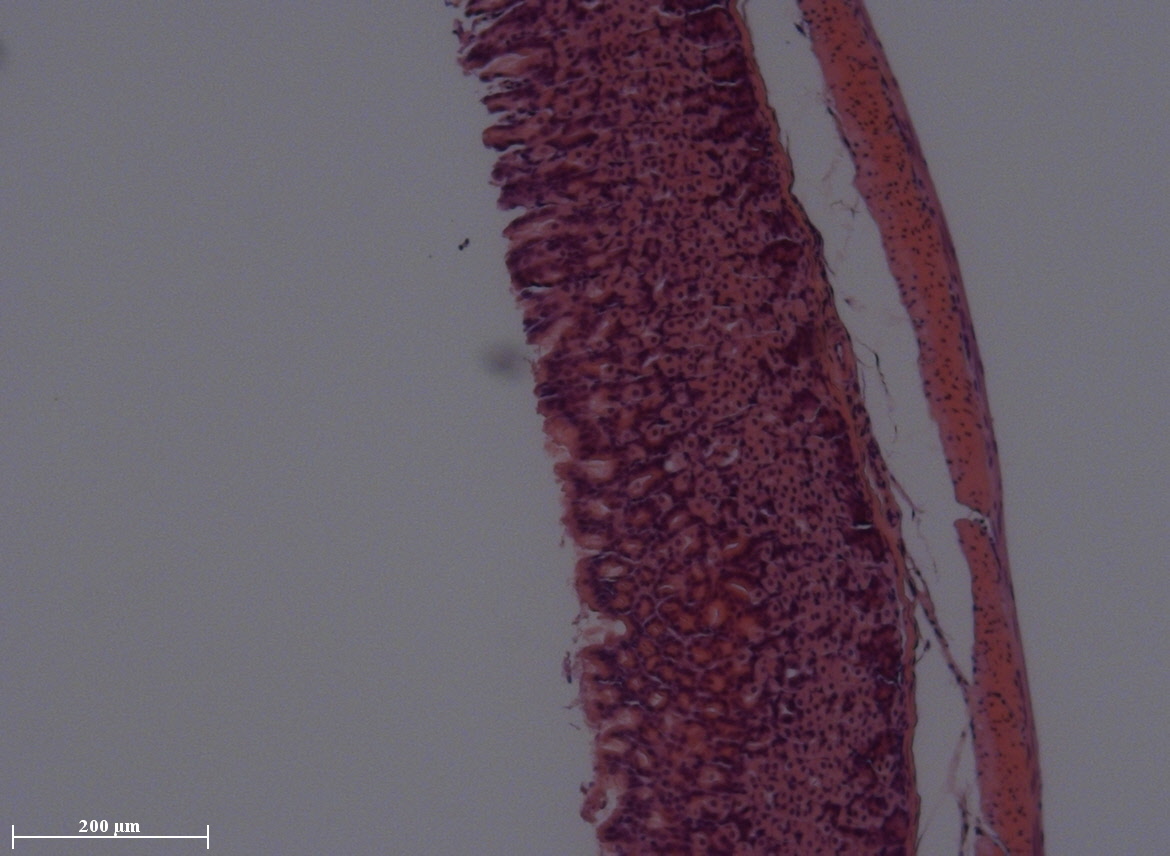

Supplement: S1 Data — (ZIP) [file pone.0131444.s001.zip › Raw data for PONE-D-14-46387R1/WT INF.JPG]

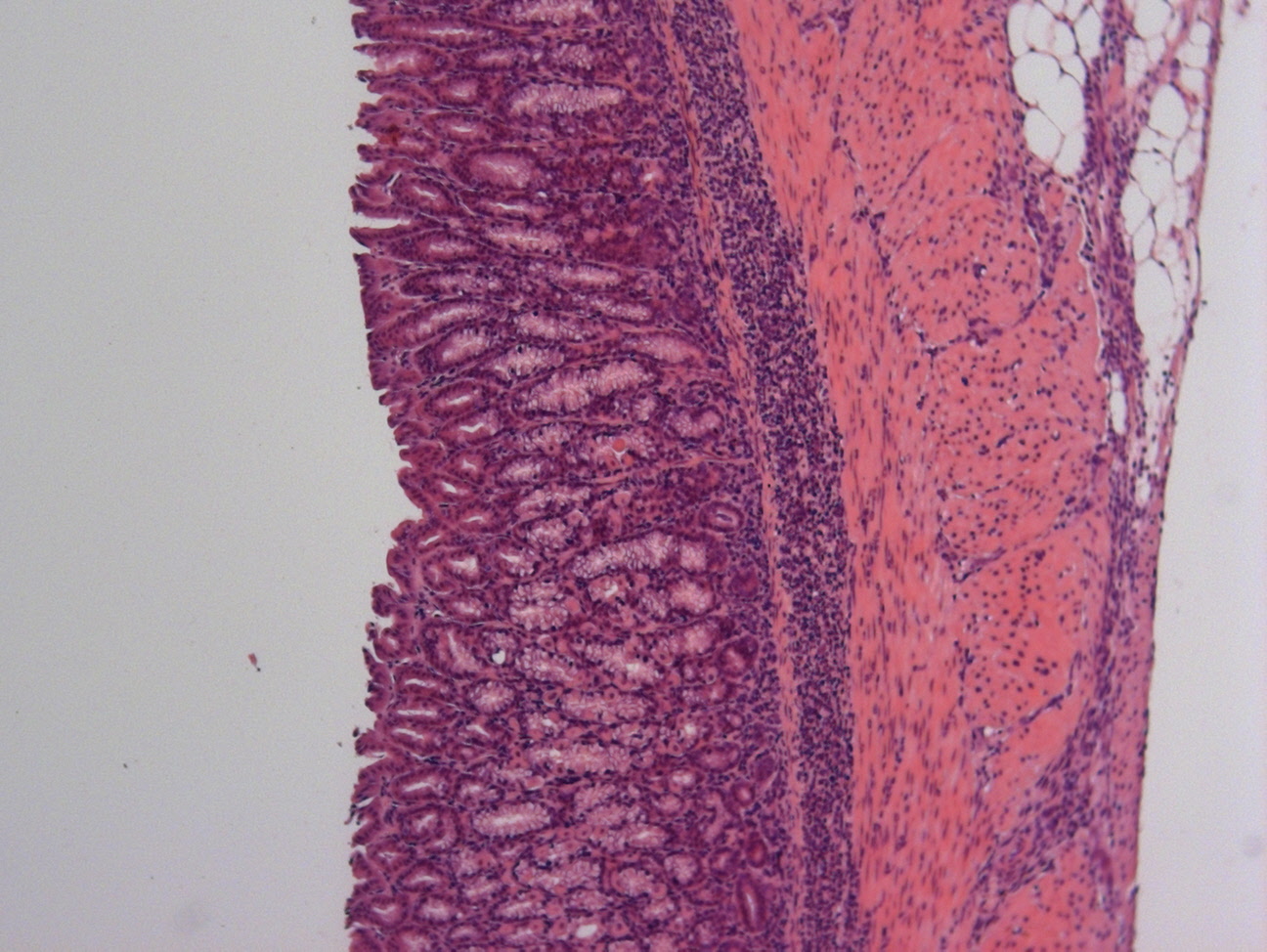

Supplement: S1 Data — (ZIP) [file pone.0131444.s001.zip › Raw data for PONE-D-14-46387R1/WT SL.JPG]
